# College and Hospital Notes

**Published:** 1905-03

**Authors:** 


### COLLEGE AND HOSPITAL NOTES.

DR. WILLIAM G. BISSELL, bacteriologist of the department of health, is delivering a laboratory course on clinical bacteriology at the dental school laboratory, University of Buffalo, 25 Goodrich street, Tuesday and Saturday evenings, at 8.30. The course began February 14, and will last six weeks. The fee is $15.00.

---

THE governors of the New York Skin and Cancer Hospital announce that Dr. L. Duncan Bulkley will give a special course of four lectures on The relation of diseases of the skin to internal disorders in the Out-Patient Hall of the hospital on Wednesday afternoons, at 4.15 o'clock, commencing March 1, 1905. The course will be free to the medical profession.

---

UNIVERSITY day at the University of Buffalo was celebrated by public exercises at the Star Theater at which Professor Adelbert Moot, of the department of law, presided and delivered a short address on the progress made and making by the university. Professor Roswell Park, department of medicine, chairman of the committee on academic extension, read an interesting report; then the principal address of the day was delivered by the Rev.

Charles C. Albertson, of Rochester, who took for his subject, Washington and Lincoln—a comparison. Music was furnished by the U. of B. Glee and Mandolin clubs. The students were present 700 strong, and friends of the university crowded the theater to its capacity. Altogether, it was a dignified and interesting occasion.

